# Children and Adolescents with Perinatal HIV-1 Infection: Factors Associated with Adherence to Treatment in the Brazilian Context

**DOI:** 10.3390/ijerph13060615

**Published:** 2016-06-21

**Authors:** Maria Letícia Santos Cruz, Claudete A. Araújo Cardoso, Mariana Q. Darmont, Paulo Dickstein, Francisco I. Bastos, Edvaldo Souza, Solange D. Andrade, Marcia D’All Fabbro, Rosana Fonseca, Simone Monteiro

**Affiliations:** 1 Infectious Diseases Department, Hospital Federal dos Servidores do Estado, Rua Sacadura Cabral 178, Rio de JaneiroRJ 20221-161, Brazil; marianadarmont@gmail.com (M.Q.D.); paulo.dickstein@gmail.com (P.D.); 2 Department of Maternal and Child Care, School of Medicine, Fluminense Federal University, Rua Marquês de Paraná, 303, Niterói RJ 24033-900, Brazil; claudetecardoso@id.uff.br; 3 Health Information, Fundação Oswaldo Cruz, Biblioteca de Manguinhos suite 229, Av. Brasil 4365, Rio de Janeiro RJ 21045-900, Brazil; francisco.inacio.bastos@hotmail.com; 4 Department of Pediatrics, Instituto de Medicina Integral Prof. Fernando Figueira, Rua dos Coelhos 300, Recife PE 50070-550, Brazil; edvaldo.es@gmail.com; 5 Department of Pediatrics, Fundação de Medicina Tropical Dr. Heitor Vieira Dourado, Av. Pedro Teixeira 25, D Pedro I, Manaus AM 69040-000, Brazil; douradosol@yahoo.com.br; 6 Centro de Doenças Infecciosas e Parasitárias de Campo Grande, Rua Bahia 280, Jardim dos Estados, Campo Grande MS 79002-380, Brazil; fabbro@uol.com.br; 7 Department of Pediatrics, Hospital Fêmina, Grupo Hospitalar Conceição, Rua Mostardeiro 17, Moinhos de Vento, Porto Alegre RS 90430-001, Brazil; rosana040@yahoo.com.br; 8 Laboratory of Environmental and Health Education, Instituto Oswaldo Cruz, Fiocruz. Av. Brasil 4365, Rio de Janeiro RJ 21045-900, Brazil; msimone@ioc.fiocruz.br

**Keywords:** patient adherence, HIV infection, children, adolescents, vulnerability, caregivers

## Abstract

Challenges to the adherence to combination antiretroviral therapy among the pediatric population should be understood in the context of the trajectories of families, their interaction with healthcare services, and their access to material and symbolic goods. The present study analyzed individual, institutional and social factors that might be associated with the caregivers’ role in the treatment adherence of children and adolescents living with HIV (CALHIV). Based on semi-structured interviews and questionnaires applied to 69 caregivers seen at pediatric AIDS services of five Brazilian macro-regions, we observed that adherent caregivers had better acceptance of diagnosis and treatment, were less likely to face discrimination and social isolation secondary to AIDS-related stigma and tended to believe in the efficacy of treatment, and to be more optimistic about life perspectives of CALHIV. Interventions aiming to improve adherence and to promote the health of CALHIV should take in consideration the interplay of such different factors.

## 1. Introduction

The key aim of HIV infection treatment is to suppress viral replication; this aim requires optimal adherence to combination antiretroviral therapy (cART) and should be monitored over time by periodical assessment of serum HIV viral load (VL). Treatment failure usually requires the prompt return to optimal adherence and/or the adoption of new therapeutic regimens in order to avert progressive immune deficiency, clinical deterioration and, eventually, death. Regarding both individual-level infection dynamics and the health of the public, non-adherence to treatment favors the appearance viral resistance and eventual transmission of drug-resistant HIV strains [1].

Studies on cART adherence among children and adolescents living with HIV (CALHIV) point to some barriers that involve several domains: intrapsychic, family and social relationships, service characteristics, as well as factors intrinsic to the medications themselves. These barriers include the following: difficulties of communication between parents and children, non-disclosure of diagnosis, premature attribution of the responsibility for the treatment to children themselves, cognitive deficits, high levels of stress, poor quality of life [2,3,4], adverse effects of medication, fear of discrimination and/or effective stigma, having the biological parents as caregivers, having lost one parent, and long treatment duration [4,5,6,7].

However, the results of studies on treatment adherence exhibit some inconsistencies, particularly within the pediatric population [5,8]: factors such as age, gender and education and some caregivers’ sociodemographic characteristics were found to be associated with both adherence and non-adherence [9]. These inconsistencies suggest that adherence to treatment involves nonlinear interactions among the factors under analysis and that some underlying structures of interdependence among them are not systematically considered in an adequate manner [10]. Emphasis on the interactive, dynamic nature of these factors and their interplay is key for the analysis of interventions aimed at optimizing adherence to treatment. Several studies indicate the need for multifaceted, long-lasting and flexible actions, addressing both social (stigma) and situational (service location and functioning) factors, as well as highlighting the characteristics and untoward effects associated with the different therapeutic regimens [11].

The hypothesis informing the present study was that the challenges faced by CALHIV to adhere to cART should be understood within the context of the trajectories and living conditions of the caregivers who are responsible for administering medications and their interaction with healthcare services.

Based on the aforementioned considerations, the present study sought to identify and analyze the individual/family, institutional and social factors that contribute in an integrated manner to foster a better (or worse) adherence to cART among the pediatric population. For this purpose, we first classified CALHIV seen at pediatric AIDS services as adherent or non-adherent to treatment based on biological criteria (VL testing history). Next, we compared the following characteristics of the caregivers who were responsible for administering treatment to CALHIV: history of HIV diagnosis and diagnosis communication, their socioeconomic and psychosocial profile, as well as their views and practices relative to cART, their interaction with healthcare services and concerns about HIV-related stigma.

## 2. Materials and Methods

**Figure 1:**
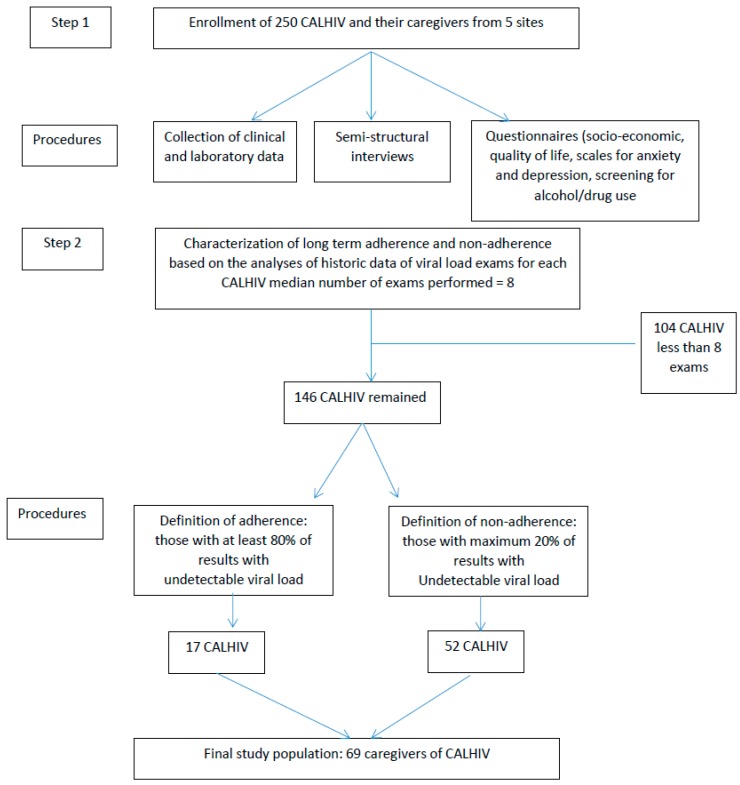
Study design and population.

The present study is part of a larger multicity project investigating the adherence to cART of CALHIV seen at five pediatric AIDS services in Brazil, located in five cities (Rio de Janeiro, Recife, Manaus, Campo Grande and Porto Alegre). The project was approved by the IRBs (CAAE 15843613.7.1001.5240) of all five participating institutions and was supported by the Department of Sexually Transmitted Diseases/AIDS and Viral Hepatitis, Brazilian Health Ministry [12]. The study used different methodological procedures described on Figure 1 and included 250 caregivers. The number of participants from each center was proportional to the total number of CALHIV seen at these facilities.

As a first step, all 250 caregivers answered structured questionnaires that included socioeconomic profile, their quality of life evaluated with the short version of World Health Organization questionnaire (WHOQoL-bref) [13], their anxiety and depression scores evaluated with the Hospital Scale for Anxiety and Depression [14], and their alcohol and drug using habits evaluated with the Alcohol, Smoking and Substance Involvement Screening Test [15]. The study abstracted clinical, laboratory and pharmacy data, including all available results of VL exams during cART, for each child. We also reviewed the situation where the diagnosis of CALHIV occurred and have stablished two categories: diagnosis due to symptomatic condition versus diagnosis as follow-up of HIV-exposed child (those born to women living with HIV). Caregivers were also submitted to semi-structured interviews aiming to better understand the participants’ life perspectives and the meaning of living with AIDS and to be under treatment. Healthcare professionals from the multi-professional staff at the five participating institutions were trained to apply the questionnaires and to perform semi-structured interviews with the 250 caregivers. The principal investigators visited each participating center to provide specific training to the staff members who were in charge of data collection with the purpose of standardizing procedures used in the survey and qualitative components. The methods and findings from the survey, including clinical profile, caregivers’ characteristics and use of cART were summarized in a previous publication [16].

Given that cART demands long-term commitment, at the second step of the study we characterized the CALHIV as “adherent” or “non-adherent” based on VL tests performed over time, since the beginning of treatment. The clinical coordination of the study defined patients with 80% or more VL serial results below the limit of detection as “adherent” and those with less than 20% of such results over time as “non-adherent”. Since exams had been collected for routine care, at this point we observed a wide variability in the number of exams performed for each participant CALHIV. Analysis of data showed that the median number of exams performed per CALHIV was 8 and we opted to exclude 104 CALHIV who had less than eight available VL results. Among the remaining 146 CALHIV, 17 met the criteria to be considered adherent and 52 non-adherent, corresponding to 69 caregivers. It was interesting to notice that there were 77 CALHIV who did not meet criteria to be considered either adherent or non-adherent. These CALHIV showed variable patterns of viral suppression, alternating VL with results below the level of detection with results showing viral replication in blood.

Most of the adherent CALHIV were from Recife, 12/17 (70%); among the non-adherent patients, 20/52 (38%) were from Recife, 15/52 (29%) were from Rio de Janeiro, 9/52 (17%) were from Manaus, 6/52 (12%) were from Campo Grande, and 2/52 (4%) were from Porto Alegre.

The present paper comprises analysis of the interviews, sociodemographical and laboratory data of CALHIV of these 69 caregivers, based on the contribution of social science approach in the comprehension of the nexus between cultural systems, social hierarchies and access to material and symbolic goods within the context of social behaviors of individuals and social groups. Our aim was to investigate intra- and inter-group similarities and differences between the 17 caregivers of the adherent CALHIV and the 52 caregivers of non-adherent CALHIV that were likely to reveal the dynamics of the factors associated with adherence to cART in this population.

After the transcription of the 69 interviews, the data analysis was organized and interpreted following four steps. The first one was exhaustive reading of the material to identify emerging themes and categories derived from the objectives of the study. The second step was the development of analytical categories based on the theoretical perspective of the research. The third step was the codification of the empirical material in order to identify the analytical categories. Lastly was the interpretation of the coded material in accordance with the study objectives, literature review and theoretical concern [17].

## 3. Results

The questionnaire data showed that the socioecomonic characteristics and the proportion of biological mothers were similar among the caregivers of the 17 adherent and 52 non-adherent CALHIV; in addition, 69% of the caregivers of adherent CALHIV and 71% of the caregivers of non-adherent CALHIV did not report to have abused alcohol or other substances. The groups differed in terms of high scores for anxiety according to standard scales (19% of caregivers of adherent CALHIV *vs*. 44% of caregivers of non-adherent CALHIV), high scores for depression according to standard scales (6% of caregivers of adherent CALHIV *vs*. 25% of caregivers of non-adherent CALHIV), and in the fact children’s diagnosis was based on their symptoms (38% of caregivers of adherent CALHIV *vs*. 48% of caregivers of non-adherent CALHIV), as opposed to those tested as follow-up of HIV-exposed children. Table 1 shows clinical characteristics of adherent and non-adherent CALHIV.

The interviews’ analysis revealed that caregivers of adherent CALHIV differ from non-adherent caregivers, regarding the following categories: acceptance of own and/or child’s diagnosis, valorization of and availability for care delivery, belief in the efficacy of treatment and survival perspectives, connection between users and healthcare team and support from family and community networks. As shown in Table 2, these categories were classified in three adherence-related dimensions as follows: (1) individual: the context of testing for HIV and the communication of diagnosis, psychological/behavioral factors and caregivers views and practices related to the benefits of cART and the life perspectives of CALHIV; (2) institutional: the characterization of CALHIV’s access to cART and of the interactions between caregivers and professionals at the pediatric AIDS services; (3) social: the nature of the (social-, family- and community-based) support received by the caregivers and putative experiences of social isolation secondary to AIDS-related stigma.

The vast majority of the 52 caregivers of non-adherent CALHIVrefer to experiences of discrimination and social isolation, which are secondary to AIDS-related stigma. These experiences contribute to the difficulty in accepting the diagnosis of HIV infection. More than a half of caregivers in this group report non-acceptance of diagnosis and the experience of suffering among family or community members, as illustrated bellow.

“*(...) it’s quite difficult because of the prejudice… I’d even accept (it). Just me. What I can’t accept it’s because my son has it and he’s a child and I could have helped if I’d known when I was pregnant. That’s the only thing that tortures me.*”

(Boy’s mother; Rio de Janeiro)

“*His mother also suffered a lot. But we didn’t know the cause of her suffering (…) She killed herself, she didn’t let the disease manifest in her.*”

In turn, most of the 17 caregivers of adherent CALHIV believe that the children need and deserve respect, affection and special care. This perspective is partly due to their view of the affected children as innocent victims and their understanding of the negative effects of AIDS-related stigma. Adherence to treatment thus becomes an opportunity to repair (or reduce) the damages associated with the infection.

“*The person who lives (with HIV) is like this, he/she has to be well understood, be well-cared for, with affection (…) So I said, I want to keep her. And she said: see, Mrs. X, are you aware of the girl’s problem? I said: indeed, that’s why I want (to take care) (…) I want it very much, more than anything else. And now I won’t give her to anyone else. It has to be me; I will take responsibility for everything.*”

(Girl’s grandmother; Recife)

The value attributed to CALHIV care, which seems to be translated into efforts to ensure their adherence to treatment, as well as family support were associated with the acceptance of diagnosis. AIDS, which is still believed to be a fatal and morally condemnable disease, has gained a new meaning for the interviewees due to the possibilities of living with HIV that are presented by healthcare professionals.

“*... when I learned about that stuff he has, I refused to accept, I cried a lot. Dr. Y gave me a lot of advice, that I should have to (…) that I should accept him the way he is, she explained me how to take care of him, but it was very difficult; it’s still difficult.*”

(Boy’s grandmother 2; Recife)

The caregivers’ belief in the quality of care delivered at the service are common to all caregivers of adherent CALHIV. Their belief in the efficacy of regular treatment to expand life perspectives of CALHIV and to preserve their rights to health and education are evident in their investment in education and their plans for the future (to have a family, to work); this attitude is illustrated by the following excerpt of a caregiver of an adherent patient:

“*So I think about him growing up, graduating (…) working, getting married (…) I wonder, will X get married? Will he have children? How will X’s daily life be? (…) I’m very insistent that he studies, to have a good job, work somewhere like a bank, something good for him.*”

(Boy’s aunt; Recife)

In our study, commitment to treatment has been frequently accompanied by taking responsibility for related actions, such as showing up for consultations, refilling prescriptions and administering medications. In some cases, such responsibility was explicitly manifested by expressions such as “not to fail, not to miss” or sentences such as “*If I fail, I’ll kill myself*”.

“*I always come, on the right date. I’ve never missed a single day. She’s been seeing Dr. W for 10 years now, and has only missed one appointment. (…) When the day comes, I’ve got to come and get the medication, I come, I take it at the right time. I never miss a date, never miss the time.*”

(Mother; Recife)

Interviews were conducted by healthcare professionals affiliated with the clinical services and their participation in the study was seen as an opportunity by the caregivers to obtain further information, to discuss the issue and to receive orientations about treatments, valued chiefly by caregivers of adherent CAVHIV.

“*This is what it can be done* (...) *help with words, with words, with strength. Words of affection, of strength, positive words. To guide about something that I don’t understand, about something that I don’t have, that I can’t do, that I can’t be (…) Thank you. You bringing me here was a pleasure (…) It was good, I even let it off my chest (…) I was all locked up here. It was good for me, I was a help (…) a good help indeed, a huge help.*”

(Girl’s grandmother; Recife)

On the opposite side, most caregivers of non-adherent CALHIV have negative thoughts about the treatment, although they rarely admit difficulties in following the prescriptions. Biological mothers frequently had troublesome experiences in taking their own medication due to the physical and mental side effects of the drugs. This is illustrated by the following excerpt:

“*You see, to take this medication (…) is awful, you know? Because it’s so many medicines (…) Sometimes I say: when I die and the doctors open me, there’ll be only pills inside me, because I take so many medicines. And then, when I take them, I feel ill (…) And when I don’t take them I don’t feel anything (…) do I really have this (disease)? Because I take it and I feel ill, an agony. I want to throw up, I throw up all day long, I feel dizzy, that kind of stuff, you know? And when I don’t take (the medication) I feel nothing (…) they said I have HIV, but I didn’t see the test (results), I didn’t see anything. What the doctors tell me to do, I do, but not all, you know? Because at times I give myself a rest.*”

(Girl’s mother; Recife)

As illustrated by the excerpt presented above, the medications used came to represent the disease itself because the interviewee was asymptomatic before she started taking the medications; in other words, from her subjective perspective, the symptoms came to acquire concrete existence and to characterize her condition as “ill” (although imprecisely) due to the use of medication. Discomfort is also related to the perception that treatment is a continuous and endless battle because the medication will not solve the problem (cure AIDS). In this context, the treatment of children tends to be more difficult than the treatment of adults due to the scarcity of therapeutic options, which are often available in poorly tolerated forms (e.g., having an unpleasant taste).

Around 40% of caregivers of non-adherent CALHIV refer at least one reason for disbelief about the positive effects of treatment on extending life expectancy and on maintaining the quality of life. The narratives of these caregivers suggest CALHIV lack future life perspectives. Such disbeliefs seem to be reflected in the caregivers’ lack of valorization of long-term investments or projects, such as education, as shown by the following excerpt of a child’s grandmother interview:

“*She’s doing well at school, but I think that she won’t have a future (…) She’s thinking of going to college, work, getting a better job, something good for her (…) she tells me: Grandma, I’ll graduate, I’ll help you. But I don’t think it will happen (…) (no matter) all she learns, all she devotes herself to, all I do for her, that everybody here does… But I don’t think she has a long life, a future. It’s just a (...) like something (she does as) entertainment so as not to just pass through life. But I know that it has no cure. The person has no future (…) I think it’s a pity. You see, you look at her so healthy in appearance, so full of life. But it’s a life (…) so short, because if she stops taking the medication, she has a short life.*”

(Girl’s grandmother; Manaus)

Among caregivers of non-adherent CALHIV it was common to observe difficulties with disclosure of the diagnosis to CALHIV and fear that the use of medication might lead to disclosure of the diagnosis to partners, friends and family members.

“*... my son will soon be a man and will want to have a family of his own, and what will happen? It will be a huge problem for him (…) I keep thinking what I’ll say when he’s 15, 16 and finds a girlfriend (…) because it’s difficult to me (…) sometimes he asks me: mom, why do we take so many medicines? I never knew how to sit down with him and explain. These things hurt me; they’re accumulating inside me (…)*”

(Boy’s mother; Rio de Janeiro)

“*To me it means much sadness, too much prejudice (…) it breaks my heart, I get sad (…) the harmful thing is not the HIV, but all the prejudice (…) it ties me down. I don’t have strength (…) Sometimes I take the medicine, sometimes I don’t, because sometimes there’s someone at home who cannot see it (…) I remove the labels from the medication, but then, they ask me why I remove the labels from the medication.*”

(Girl’s mother; Rio de Janeiro)

## 4. Discussion

The study has a multi-step design developed to unable identification of caregivers’ and CALHIV’s commitment to treatment, based on biological criteria (VL testing history). We recognize that the rigor of selection process that excluded those CALHIV who had less than 8 available VL results during follow-up may have created a bias, since those with less exams are more likely to be non-adherent. Nevertheless, we have tried to compensate the possible exclusion of the most non-adherent patients defining that among the remaining CALHIV, those with less than 20% of exams showing viral suppression should be considered as non-adherent.

Although the socioeconomic profile and access to pediatric AIDS services were similar among the 69 caregivers of CALHIV under analysis, in all five investigated cities, a comparative analysis between the 17 caregivers of the adherent and the 52 caregivers of non-adherent CALHIV (defined after biological criteria, viral load results) showed how individual, institutional and social factors interactively influence cART adherence.

Twelve of the 17 caregivers of adherent CALHIV were from the same center in Recife. The Recife center, like those in Manaus and Campo Grande, serves the metropolitan population and is a reference center for the treatment of CALHIV from neighboring towns and rural areas. Among the five participating centers, the center in Recife stood out as providing care to the largest number of CALHIV and consequently, for recruiting the largest proportion of participants in this study. This good result might be associated with the wide experience of the staff in the management and care of pediatric patients and their families. However, this hypothesis needs to be further investigated in future studies to achieve a better understanding of the role that healthcare services play in CALHIV treatment adherence.

In regard to the history of HIV diagnosis, the adherent CALHIV were, in general, diagnosed during the process of investigating the medical and family histories of seropositive parents; whereas the non-adherent CALHIV were tested due to the presence of symptoms suggestive of HIV infection. HIV diagnosis delay was found to be a major influence on treatment adherence, reinforcing the relevance of prenatal care and programs that aim to prevent HIV vertical transmission as a first, key intervention to improve treatment adherence of infected children. These families might have greater opportunities to develop relationships with healthcare staff and to commit themselves to the necessary care. However, even in cases where this first opportunity is lost, later interaction between CALHIV/relatives and healthcare staff might contribute to redefine their fatalistic perspective of AIDS, with a consequent broadening of the life perspectives of people living with HIV and their families.

A study conducted at an AIDS service in Rio de Janeiro found that children diagnosed due to disease manifestation in the first months or years of life might be undervalued and discriminated against by their families. The study also made evident that some families are afraid of disclosing the diagnosis to CALHIV and secrecy may become an additional source of discrimination. Some CALHIV present a slow evolution of the infection, and diagnosis might be delayed for many years, which might slow the assumption of their new identity. Last but not least, the study also documented CALHIV who grow up in institutions may face difficulties to adapt to their families when they return home [18].

Fear of AIDS-related stigma might influence the acceptance and disclosure of HIV diagnosis among the population under study. Excerpts from caregivers’ narratives suggest that among caregivers of non-adherent CALHIV failure to cope with AIDS diagnosis might be secondary to the internalization of a deteriorated social identity due to the associated stigma [19]. Thus, a perverse circle may emerge, whereby the fear of being rejected is fed by experiences of social isolation and a lack of social and family support, which in turn contribute to the non-acceptance and non-disclosure of the diagnosis. A meta-analysis of studies carried out in 2002–2012 on the impact of HIV on the quality of life of adults (3348 participants, assessed based on WHOQoL-BREF or WHOQoL-HIV-BREF) found that the lowest scores were related to the “social relationships” domain. This finding was attributed to the interference of stigma and discrimination within relationships with family members, friends and coworkers [20].

The communication of an HIV diagnosis to children and adolescents may represent a challenge, especially in low-income countries. A review of studies published from 1997 to 2008 showed that social, economic and racial disadvantages are associated with a greater difficulty in disclosing such a diagnosis due to a greater concern about stigma and discrimination [21]. Although caregivers are aware of the potential benefits of communicating the diagnosis, this communication is often delayed based on the idea that children are immature or are not interested in the matter [8]. In addition, some caregivers express the fear that the children might tell other people about their condition, and they have a sense of guilt for having transmitted the infection to their children. 

Among the caregivers of adherent patients, the predominant attitude was one of acceptance of the status of living with HIV and a firm belief in the patient’s high survival chances and future perspectives, once optimal treatment adherence is achieved. These findings reiterate that disclosure of the HIV diagnosis to children should be a component of care provided to this population of patients because it is associated with positive outcomes regarding treatment adherence and health status. A systematic review of studies on cART adherence showed that high esteem and the acceptance of a seropositive status facilitate treatment adherence [22]. Additionally, a qualitative study with 17 caregivers of CALHIV aged zero to 18 years old that was conducted in Belgium found that caregivers who accept the diagnosis of disease tend to better incorporate useful information and are more motivated to fight for the child’s health [6].

The establishment of good interactions with the healthcare staff and their trust regarding the benefits of proper treatment adherence has been identified as a key element of optimal adherence. In contrast, families of non-adherent patients had limited relationships with healthcare professionals and usually mistrust the potential benefits of treatments for CALHIV’s survival, and consequently did not redefine their fatalistic worldviews. These caveats usually originated from experiences of social isolation and lack of social, family and community support, which are associated with AIDS-related stigma. These findings show that in addition to providing access to diagnosis and treatment, it is also necessary to address the social exclusion of people living with HIV/AIDS and their relatives (which is caused by AIDS-related stigma as well as precarious living conditions) by means of sound partnerships between institutions, patients and their families [23].

Finally, attempts at achieving satisfactory adherence to treatment among CALHIV should consider the dynamic nature of the process and the life stories of the families living with HIV. The main strength of our study is the combination of direct (biological effects) and indirect (questionnaires, interviews) methods of assessment treatment adherence [24]. In addition to distinguishing itself from the cross-sectional approach that measures treatment adherence in an exclusively dichotomous manner (adherent *vs*. non-adherent), which is predominant in the treatment adherence literature, the approach used in the present study enabled the consistent identification of groups of caregivers of adherent or non-adherent CALHIV and to analyze their characteristics and mutual differences.

This comparison has self-evident limitations. The considerable difference in the size of the two groups (adherent and non-adherent) did not allow the analysis of characteristics with relatively low frequencies. Nevertheless, the characterization of the individual, institutional and social factors that determine the role that caregivers play in the adherence of CALHIV to cART showed how these factors act in a complementary manner within a complex articulation regarding the dynamics of adherence or non-adherence to treatment. Thus, despite the similarities in their socioeconomic profile and access to reference services, we detected differences among the families that proved to be associated with CALHIV’s adherence/non-adherence to treatment. By contributing to a more thorough understanding of the dynamics involving the factors involved in cART adherence in the pediatric population, this study provides useful information that can guide a revision of the practices performed at pediatric AIDS services.

## 5. Conclusions

Pediatric AIDS healthcare teams should anticipate possible hindrances to treatment adherence and seek to minimize them. Our findings call attention to dynamic situations in which individual, institutional and social factors should be worked out together with the involved families based on dialogue, active listening and facilitating caregivers, children and adolescents to overcome the conditions that keep them vulnerable to non-adherence to treatment [25].

In addition to improving the interaction between families and healthcare services, the outcomes of the treatment of CALHIV might benefit from interventions that target cultural and structural aspects. Participation of the community in dealing with AIDS-related stigma is an integral part of strategies that seek to overcome obstacles and promote human rights and effective changes in the legislation and discriminatory practices [26]. Within this context, the process of the primary socialization of CALHIV must be a focus of attention to prevent the next generation from being affected by HIV-related stigma.

## Figures and Tables

**Table 1:** Clinical characteristics of adherent and non-adherent CALHIV receiving antiretroviral treatment in five centers in different geographic regions of Brazil.

Characteristics	Adherent (*n* = 17)	Non-Adherent (*n* = 52)
Male gender (%)	59%	58%
Mean age at study participation (y)	11.6	11.2
In use of first ART regimen (%)	53%	33%
Had AIDS defining condition at treatment beginning (%)	53%	39%

**Table 2:** Categories that differentiate caregivers of adherent and non-adherent CALHIV based on interviews with caregivers of CALHIV receiving antiretroviral treatment in five centers in different geographic regions of Brazil.

	Individual	Institutional	Social
Caregivers of adherents (*N* = 17)	Greater acceptance of own and/or child’s diagnosis Valorization of and availability for care delivery Belief in the efficacy of treatment and survival perspectives; invests in the child’s future (e.g., education) Acknowledgment of the relevance of disclosing the diagnosis to the child/adolescent Commitment to drug administration	Greater connection (exchanges) and dialogue between users and healthcare professionals resulting in the commitment of both Interaction between healthcare teams and therapeutic resources contributes to redefine the fatality of AIDS and enlarge the life perspectives of people living with HIV/AIDS (PLHIV)	Receives support from family and community networks Does not experience social isolation due to AIDS-related stigma
Caregivers of non-adherents (*N* = 52)	Difficulty to accept own and/or the child’s diagnosis Feelings of guilt for having transmitted HIV to the child Limited belief on ART effects and life and future perspectives Difficulty to disclose the diagnosis to the child/adolescent Difficulties to tolerate own treatment	Less connection (exchange) between users and healthcare professionals Limited interaction between professionals and users does not favor redefining the fatality of AIDS and contributes to the lack of life perspectives of PLHIV	Fragility of the potential family and community network support Experiences of social isolation due to AIDS-related stigma

## Abbreviations

The following abbreviations are used in this manuscript:

cART	combined antiretroviral therapy
VL	viral load
HIV	Human immunodeficiency virus
CALHIV	children and adolescents living with HIV
AIDS	Acquired Immunodeficiency Syndrome
IRB	Institutional Review Board
WHOQoL-HIV-BREF	World Health Organization instrument for evaluation of quality of life among people living with HIV (short version)
PLHIV	person living with HIV
